# Solid-Phase Extraction of Polar Benzotriazoles as Environmental Pollutants: A Review

**DOI:** 10.3390/molecules23102501

**Published:** 2018-09-29

**Authors:** Ida Kraševec, Helena Prosen

**Affiliations:** Faculty of Chemistry and Chemical Technology, University of Ljubljana, Večna pot 113, SI-1000 Ljubljana, Slovenia; ida.krasevec@fkkt.uni-lj.si

**Keywords:** benzotriazoles, solid-phase extraction, environmental samples

## Abstract

Polar benzotriazoles are corrosion inhibitors with widespread use; they are environmentally characterized as emerging pollutants in the water system, where they are present in low concentrations. Various extraction methods have been used for their separation from various matrices, ranging from classical liquid–liquid extractions to various microextraction techniques, but the most frequently applied extraction technique remains the solid-phase extraction (SPE), which is the focus of this review. We present an overview of the methods, developed in the last decade, applied for the determination of benzotriazoles in aqueous and solid environmental samples. Several other matrices, such as human urine and plant material, are also considered in the text. The methods are reviewed according to the determined compounds, sample matrices, cartridges and eluents used, extraction recoveries and the achieved limits of quantification. A critical evaluation of the advantages and drawbacks of the published methods is given.

## 1. Introduction

Solid-phase extraction (SPE) is one of the most frequently used techniques for sample preparation in environmental analysis, not only for well-known organic pollutants, but also for contaminants of emerging concern. The latter group includes polar benzotriazoles, which are the focus of this review. We aim to present an overview of the SPE methods applied for the determination of benzotriazoles in aqueous and solid environmental samples, as well as in several other matrices. The literature reviewed in this work has been published in the last decade (2008–2018).

In general, SPE is a procedure in which the dissolved analytes are put into contact with a solid phase, where they are retained due to the interactions with the sorbent. Ideally, the matrix of the sample solution remains unaffected by the sorbent, and solely the analytes are extracted from the sample. The selectivity of the extraction is mainly determined by the sorbent, which can be chosen among reversed-phase (C${}_{18}$), normal-phase (silica), ion exchange and mixed-mode (polymeric) phases. Moreover, in the many decades of SPE use, various forms of sorbents have been developed, for example sorbents in the shape of disks, cartridges and well plates. The principal difference between these configurations is their capacity, which is directly related to the mass of the contained sorbents. In environmental research, cartridges are the prevalent form of SPE sorbents.

Generally, an SPE method consists of four steps, usually performed with the aid of a vacuum manifold. The cartridge is first conditioned with an organic solvent and pH-adjusted water solution. This minimizes the surface tension of the sorbent (especially important in reversed-phase sorbents) and enables interactions with the desired form of sorbents (protonated or de-protonated in ion exchange and mixed-mode sorbents) [1]. Secondly, the sample is loaded onto the sorbent and a washing or rinsing step is performed. This facilitates the removal of weakly bound interferences (matrix components) and strengthens the analyte–sorbent interactions. Finally, the sorbent is dried under vacuum or under nitrogen flow and the analytes are eluted with an organic solvent optimized to sever the analyte–sorbent interactions (usually pH-adjusted in ion exchange and mixed-mode sorbents).

## 2. Benzotriazoles

Benzotriazoles are heterocyclic compounds, with the basic structure of a benzene ring fused to a triazole ring. Non-polar benzotriazoles, usually hydroxyphenyl derivatives, are used as UV stabilizers in plastic materials and personal care products [2]. Polar benzotriazoles, including the basic benzotriazole and the methyl-, hydroxy- and chloro- derivatives, are used as corrosion inhibitors in antifreeze liquids, various industrial systems (cooling, braking, cutting and metal-working fluids), aircraft de-icing fluids and household dishwashing detergents [2,3,4]. The most commonly used are the benzotriazole (BTZ) and a mixture of 4- and 5-methyl-benzotriazole (MBZ) isomers, also known as tolyltriazole.

In this review, we focus on the polar compounds listed in Table 1. Due to a high aqueous mobility stemming from their polarity (observable as log*K*_ow_ in Table 1) they are ubiquitously present in the hydrosphere, having been found in snow, groundwater, rivers, lakes, seas, treated and untreated wastewater, recycled water and also in drinking water [2,5,6,7,8,9,10,11]. Furthermore, they have been also determined in air, soil, sediments, house dust and textiles [12,13,14,15,16], in human urine, amniotic fluid and plants [11,17,18].

The main sources of these pollutants appear to be airport run-off and industrial and municipal wastewater [2,5,8], as benzotriazoles are not readily biodegradable. According to different sources, only 13–62% of BTZ and 11–72% of MBZ are removed in public wastewater treatment plants [2,5,19] due to their long half-lives [20], with 5MBZ being much more susceptible to degradation than 4MBZ. The compounds can also pass through common steps in the preparation of drinking water [21], but can be removed from recycled water with additional treatment steps, such as reverse osmosis after ultrafiltration [22], ozonization [23] or photodegradation with UV/H${}_{2}$O${}_{2}$ [24]. Degradation pathways in activated sludge are not well known, but the suggested metabolites of aerobic digestion appear to include phenol, phthalic acid, 1-methyl benzotriazole, 4-methoxy-1*H*-benzotriazole and 5-methoxy-1*H*-benzotriazole for BTZ, 2,5-dimethyl benzoxazole and benzotriazole for 5MBZ and 5-chloro-2-methyl benzoxazole and benzotriazole for ClBZ [20].

The benzotriazoles have been shown to be toxic to aquatic bacteria, plants and invertebrates [25]. BTZ is also a possible carcinogen [6] and expresses anti-estrogenic activity [26].

As these compounds are classified as emerging pollutants, the limits for their acceptable concentrations in the environment are not generally set. The only exceptions we found in the literature are the limits of 5 $\mathrm{mg}/m^{3}$ BTZ in workplace air and 0.1 mg/L BTZ in drinking water from Russia, cited by Pervova et al. [27], and the limit of 7 ng/L 5MBZ in drinking water of Australia, reported by Janna et al. [6].

Due to low environmental concentrations, the determination methods use sensitive chromatographic techniques, such as liquid or gas chromatography with mass spectrometric detection (LC-MS, GC-MS) in concert with various extraction techniques. Along with the classical SPE, newer techniques have also been applied for their determination, for example QuEChERS (Quick, Easy, Cheap, Effective, Rugged and Safe extraction) [28], stir-bar sorptive extraction (SBSE) [29], solid-phase microextraction (SPME) [30,31] and dispersive liquid–liquid microextraction (DLLME) with optional concurrent derivatization [32,33]. In some cases, determination without pre-concentration has also been reported for samples with higher analyte concentrations [2,22], but the vast majority of the studies make use of SPE to achieve a pre-concentration of analytes and a reduction of matrix effects.

For readers with further interest in benzotriazoles, we suggest a dedicated review on toxicity and occurrence of BTZ and 5MBZ, which has been published by Alotaibi et al. [34], and a review of available analytical methods, published by Herrero et al. [35]. In contrast to these works, this review focuses only on SPE as the sample preparation technique for determination of polar benzotriazoles and also considers the research, published in the years following the publication of the previous reviews.

## 3. Analytical Techniques for the Determination of Polar Benzotriazoles

In environmental chemistry, the determination of benzotriazoles is commonly done with chromatographic methods. Due to the compounds’ polarity, low volatility and occurrence in low concentrations, the most commonly chosen techniques are LC for separation and MS for sensitive detection and identification. Occasionally, UV/VIS or fluorescence detection [32,36] have also been used, but noticeably higher limits of quantification (LOQ) have been achieved in those cases.

In the majority of cases, separation has been performed on various C${}_{18}$ columns [4,10,11,21,37,38,39], although columns with phenyl stationary phase have also been used [2,8,22]. Usually, the mobile phase has initially a high water content and is then gradually adjusted to raise the ratio of organic solvent, this solvent being either methanol (MeOH) [10,40], acetonitrile (ACN) [22,32] or both [38,41]. The critical problem of the LC is the separation of 4- and 5-methyl-benzotriazole isomers, due to their similar retention. It has been claimed that the use of ACN is necessary for their separation [35] and in the cases where both solvents were tested, better chromatographic profiles and lower retention times [32] or better peak heights and shapes [39] were achieved when using ACN. When LC is coupled with MS, formic or acetic acid is added to the mobile phase to increase the ionization efficiency of the analytes [4,8,21,41].

Ionization is almost invariably performed in the positive mode of electrospray ionization (ESI+), since atmospheric pressure chemical ionization (APCI) was proven to be much less sensitive, resulting in higher limits of detection (LOD) [22]. To achieve high sensitivity, tandem MS (MS/MS) or high resolution MS (HRMS) are commonly used. MS/MS is usually performed with a triple quadrupole analyser in selected reaction monitoring (SRM) mode, with two transitions being recorded for each analyte to satisfy the requirements for quantification and identification [10,42]. For HRMS, performed with Orbitrap [21,37,38] or time-of-flight (TOF) [22] analysers, only a precursor and a product ion are required for a proper identification of the analytes. The main drawback of LC-MS methods is the appearance of the matrix effect, which is the suppression or enhancement of analyte ionization due to the sample matrix. This effect depends on the matrix composition of the injected sample and the concentration level of analytes, and is often more pronounced at lower concentration levels. It is one of the main reasons for using intensive sample clean-up procedures. Other methods of circumventing this problem include the use of matrix-matched calibration or an isotopically labelled internal standard [35].

For quantification of benzotriazoles in environmental samples, external calibration [4,11,21,38,43], matrix-matched calibration [17,39,42] and the standard addition method [8,23] have been used to date. External calibration is generally used in cases of simple samples and very low matrix effects, while standard addition and matrix-matched calibration are used in the case of more complex samples, since they compensate for some of the matrix effects.

In comparison to LC, GC is used less often. Although polar benzotriazoles are quite non-volatile, they can be determined either with derivatization (trimethylsulfonium hydroxide (TMSH), acetylation [33,44,45]) or without [7,46], to reduce the complexity of the method [47]. The stationary phase used is 5%-phenyl-methylpolysiloxane or equivalent [5,7,45,46,48,49,50], except in rare cases, for example [51], where the authors tested various ionic liquids as stationary phases and reported faster separation and better peak shape than with conventional columns. A two-dimensional GC × GC-TOF method has been developed by Jover et al. [47], who separated co-eluted analytes and achieved LODs similar to LC-MS/MS.

Almost invariably, MS [5,45,49,50,52] or MS/MS [7,46] detectors are used after separation. In one case, isotope ratio MS was also performed, but the authors reported difficulties with low sensitivity, low volatility, and interactions of analytes with the exposed metal components of the instrument, demanding instrument modification or derivatization of the analytes [44]. The main drawbacks of GC-MS appear to be poor retention on non-polar columns [51], peak tailing or peak disappearance on polar columns and low sensitivity due to low *m/z* of the target ions [47].

## 4. Solid-Phase Extraction of Benzotriazoles from Aqueous Samples

As mentioned before, SPE is the prevalent extraction technique for polar benzotriazoles in environmental samples due to its high pre-concentration factors (large sample volume), smaller amounts of solvent used (in comparison to liquid–liquid extraction, LLE), practical simplicity and the possibility of automatization. The methods developed in the last decade for determination of benzotriazole pollutants in aqueous environmental samples have been collected in Table 2. In addition to the sorbent phase used, several other parameters are presented. The choice of the elution solvent can depend on recoveries and the selected analytical technique (for example volatile solvents for GC), although the elution solvent is often evaporated and the sample reconstituted in a suitable solvent (this can also serve as an additional concentrating step). Where possible, recoveries and LOQs were added as quality control parameters, as reported by the authors. Usually, the recoveries were calculated comparing the instrument response of a post-extraction spiked sample to a pre-extraction spiked sample, either in river waters or in effluent wastewater (when the former was not available). LOQs are given as reported by the authors, but it is necessary to consider that they also depend on the analytical techniques used for determination. Some of the studies reported in the Table 2, are discussed here more in-depth, as their authors presented novel research and optimization of SPE procedures (pH of the sample solution, reduction of matrix effect, sorbent phases used, etc.).

It is worth noting that in most of the reviewed analytical methods, benzotriazoles are not the only compounds being determined. Often they are the subject of a combined analysis with benzothiazoles, benzenesulfonamides or UV stabilizers [4,7,38,47], or some of them are targets of screening methods that include tens or hundreds of other micropollutants (pesticides, pharmaceuticals, personal care products, etc.) [53,54]. Naturally, this leads to the development of methods that are not perfectly optimized for all the determined compounds, as can be observed for example in recoveries of polar benzotriazoles ranging from 22 to 112% in [54]. In some of the reviewed papers, there is also very little to no emphasis on method optimization, since they focus more on the application and interpretation of concentrations and their changes in various environmental samples [5,6,52,55,56,57,58].

Considering the relatively high polarity and weakly acidic character of the polar benzotriazoles (Table 1), the relevant extraction phases for their determination include polymeric balanced polar/non-polar (HLB, Strata-X, Bond Elut PPL), strong anion (Oasis MAX, Strata-X-A) and cation exchange (Oasis MCX, Strata-X-C) sorbents (Oasis series by Waters, Strata series by Phenomenex, Bond Elut by Agilent), while C${}_{18}$ and charcoal based sorbents have been shown to be much less effective in retention than HLB [7]. As can be seen in Table 2, the most widely used cartridge sorbent is the HLB polymeric sorbent, a *N*-vinylpyrrolidone divinylbenzene copolymer with good retention for non-polar and neutral polar compounds. In most cases [5,6,7,10,21,44,47,52] the samples were acidified to pH 2–3. Some authors claim that the sample pH had great effect on recovery [10,21], shifting from less than 50% at pH 7, to 79–110% at pH 2 [7]. This is countered by authors claiming that pH had no significant effect on recovery, but the ionization suppression was higher at pH 3 than at pH 7 [4], or that the effect of pH on BTZ recovery was very low for HLB and various other sorbents (Oasis MAX, MCX, WAX, WCX, Strata-X, X-A, X-AW, X-C, X-CW) [23].

In the cases where the research focuses on the extraction method, the main aim of development is usually a reduction of matrix effects, not higher recoveries. One of the steps that has been taken to achieve additional clean-up of the samples, was the use of a Florisil filled cartridge directly connected to the HLB cartridge. In this way the SPE eluent came directly into contact with the Florisil sorbent (magnesium silicate) which adsorbed the interferences, thereby reducing the matrix effect in spiked wastewater from −65 to −40% without Florisil to −5 to −15% with Florisil [4].

A mixed-mode sorbent, which could also reduce matrix effects, has been applied for benzotriazoles for the first time by Carpinteiro et al. [8]. Oasis MAX cartridges, containing strong anion exchange and reversed-phase sorbent, were eluted with the same organic solvent mixture (MeOH/ACO) as Oasis HLB. In comparison to HLB, MAX cartridges produced cleaner extracts and lower matrix effects, which lead to the conclusion that interfering matrix components (for example humic, fulvic acids) remained retained with anionic interaction. Although the sample pH was not modified, the recoveries ranged 96–102% in river water and 82–86% in effluent wastewater, but only 50–55% in influent wastewater. These recoveries correlated with the reported matrix effects: none for river water, small suppression for effluent wastewater, and substantial suppression for influent wastewater, which was also the most complex of the investigated matrices.

The mechanisms of sorption in the mixed-mode phases have been studied by Salas et al. [38] in both Oasis MAX and MCX cartridges. The authors optimized extraction procedures for both sorbents, each including two rinsing steps. The first rinsing step consisted of pH-adjusted water, to wash off weakly adsorbed hydrophilic compounds and increase ionic interactions, while in the second step they used MeOH to rinse neutral compounds retained by hydrophobic interactions. With MCX cartridges, the analytes remained adsorbed during washing, even though the sample pH was adjusted to pH 3 and the analytes were in their neutral forms. When increasing the volume of MeOH used for rinsing, out of the 5 benzotriazoles investigated only ClBZ washed off, indicating the weakest adsorption. In MAX they noticed no differences in retention at pH 7 and 11.5, although the analytes were de-protonated at the higher pH. This led to conclusion that the mechanism of retention in both phases is through induced dipole-ionic interactions, possible due to the delocalized electron density and electronegative elements in the benzotriazole rings. Rinsing lowered the amount of interferences in the extracts and resulted in very low ionization suppression in influent wastewater extracts, while achieving high recoveries with both cartridges (61–92%).

Although higher recoveries have been reported to be achieved with HLB (more than 90%) than with MAX at pH 10 (20–64%) in wastewater [4], these examples state that to the contrary, high recoveries can be achieved also with mixed-mode cartridges.

An innovative tandem SPE method for determination of analytes of various properties (BTZ among them) has been developed by Deeb and Schmidt [23]. The Oasis MAX and MCX cartridges were connected consecutively for the sample loading and then separated for washing and elution. All the analytes in the combined extract had recoveries more than 90%, while the matrix effects were lower than 13% for all matrices, including wastewater, implying that good clean-up was achieved for all samples.

Layered ’mixed bed’ cartridges have also been used for determination of a broad range of compounds, including benzotriazoles [54,56,59,60,61], with the aim of extracting hydrophobic, hydrophilic, anionic and cationic compounds in one step. The lab-prepared layered cartridges contained a layer of Oasis HLB sorbent on the top, to adsorb compounds with non-specific interactions, and a layer of Strata-X-AW, Strata-X-CW and Isoelute ENV+ mixture on the bottom, to adsorb the remaining compounds either by ionic or hydrophilic interactions. The eluents used were acidified and alkaline organic solvents, MeOH, ACN or ethyl acetate (EA). Osorio et al. [54] reported achieving lower matrix effects with the mixed bed cartridges in comparison to the HLB sorbent (which is critical for reducing the amount of co-elutions in the LC) and higher recoveries in comparison to the MAX sorbent.

## 5. Solid-Phase Extraction of Benzotriazoles from Solid and Other Samples

SPE can be also used for clean-up of solid sample extracts obtained from sediments, activated sludge, soil, plant and detergent matrices, as presented in Table 3. The original samples have been either simply dissolved [6,44], or extracted with liquid–solid extraction (LSE) with ultrasonication, accelerated solvent extraction (ASE [14]), pressurized hot water extraction (PHWE [75]) or microwave extraction (MAE [76]).

In addition to environmental samples, polar benzotriazoles have also been determined in other samples, presented in Table 4. The analysis of detergents and industrial fluids is important for investigations of the point of origin of these compounds in the environment, while an estimation of human and foetal intake of benzotriazoles can be made from the concentrations found in urine and amniotic fluid.

Because SPE is such a staple in analytical chemistry, it is rarely compared to other extraction techniques any more, but Asimakopoulos et al. [17] have compared extractions with two SPE cartridges (Strata-X-CW and Oasis HLB) to LLE with ACN/DCM for benzotriazoles in human urine. While recoveries were acceptable with both methods (92–127% SPE with HLB, 84–90% with LLE), the researchers noted high background contamination levels in LLE and also a co-elution of an isobaric component with BTZ, which led to higher LOD (1.17 ng/mL with LLE vs. 0.38 ng/mL with SPE). Suppression of ionization was noticed for both methods; but in real samples, lower concentrations of MBZ and DMBZ have been determined when using the LLE method than with SPE.

## 6. Conclusions

Although in recent years the trends toward miniaturization of extraction techniques and an overall greener approach have led to the development of various new analytical approaches, SPE remains the mainstay of environmental chemistry. It is one of the first choices for researchers when encountering organic pollutants and this stays true also for emerging contaminants such as benzotriazoles. In recent years, many methods have been developed for the determination of polar benzotriazoles, not only for aqueous but also for solid environmental, industrial and biological samples, as presented in this review. The general trends and advances in SPE seem to be focused on reducing the matrix effects and investigating various newer sorbent phases, which is reflected also in the study of benzotriazoles, where quite a few new studies were done with mixed-mode sorbents. Benzotriazoles are also often included in screening studies of environmental samples, where the trend of more and more analytes being determined in one analysis can be observed. The amount of publications found in this corner of environmental chemistry leads us to conclude that despite its long tradition, SPE has many uses for research left.

## Figures and Tables

**Table 1:** Table of the reviewed compounds, their abbreviations and selected chemical properties (log*K*_ow_, p*K*_a_: predicted values from the Scifinder database).

Abbreviation	Full Name	log*K*_ow_	p*K*_a_
BTZ	1*H*-benzotriazole	1.44	8.38
1MBZ	1-methyl-1*H*-benzotriazole	1.08	–
2MBZ	2-methyl-2*H*-benzotriazole	1.59	–
4MBZ	4-methyl-1*H*-benzotriazole	1.82	8.74
5MBZ	5-methyl-1*H*-benzotriazole	1.98	8.74
DMBZ	5,6-dimethyl-1*H*-benzotriazole	2.28	8.92
24DMBZ	2,4-dimethyl-2*H*-benzotriazole	1.96	–
ClBZ	5-chloro-1*H*-benzotriazole	2.13	7.46
NBZ	5-amine-1*H*-benzotriazole	0.40	9.61
1OHBZ	1-hydroxy-benzotriazole	0.69	7.39
4OHBZ	4-hydroxy-benzotriazole	0.80	7.25

**Table 2:** SPE methods reported for polar benzotriazoles in aqueous environmental samples (eff–effluent, ww–wastewater, ND–no data).

Compounds	Sample Matrices	Cartidge Type, mg	Elution Solvent	Recovery	LOQ (ng/L)	Reference
BTZ, 4MBZ, 5MBZ, DMBZ, 1OHBZ, ClBZ	tap, surface, effluent	Oasis HLB 500	3×2.5 mL ACN/MeOH (1:1)	57–125%	2–11	[21,37]
BTZ, 4MBZ, 5MBZ, DMBZ	river, influent, effluent	Strata-X	5 mL EA	85–115%	31–99	[47]
BTZ, 4MBZ, 5MBZ	river	Bond Elut PPL 200	2 mL ACN/MeOH (1:1)	49–79%	6–15	[5]
BTZ, 1MBZ	groundwater, river	Oasis HLB 200	6 ml MeOH	47–56%	1	[62,63]
BTZ, 4MBZ, 5MBZ	groundwater	Oasis HLB 200	7 mL MeOH/ACO (6:4)	95–113%	50	[2]
BTZ, 5MBZ	tap, river, influent, effluent	Oasis HLB 500	5 mL 3% MeOH in DCM	101–118% (eff)	0.2	[6]
BTZ, 5MBZ	tap, groundwater, influent, effluent	Oasis HLB 500	3×2 mL DCM/MeOH (1:1)	79–108%	9.8–36.7	[7,46,48]
BTZ, 5MBZ	river, sea	Oasis HLB 500	15 mL MeOH	69 ± 10% (sea)	0.4–1.2	[40]
BTZ, 5MBZ, DMBZ	river, influent, effluent	Oasis MAX 150	5 mL MeOH/ACN (7:3)	87–99%	2–5	[8]
BTZ, 5MBZ, DMBZ	influent, effluent	Strata-X 100	10 mL hexane/EA (1:1)	78–98% (ww)	60–810	[51]
BTZ, 1MBZ, 4MBZ, 5MBZ	groundwater, river, effluent	Oasis HLB (10) + Strata-X-AW(1.4)/ Strata-X-CW(1.4)/ Isoelute ENV+(2.1)	0.1% HCOOH in MeOH	94–124%	0.2–4.2	[59]
BTZ, 5MBZ, DMBZ, 1OHBZ	river, effluent	Strata-X 200	2×5 mL MeOH/ACN (1:1)	73–104% (ww)	0.42–10	[39]
BTZ, 4MBZ, 5MBZ, DMBZ, ClBZ	river, influent, effluent	Oasis HLB 150 + Florisil 500	2×3 mL MeOH	85–93%	2	[4]
BTZ, 5MBZ	sea	Oasis HLB 200	6 mL MeOH	67–75%	0.11–0.17	[53]
BTZ, 5MBZ	effluent	Oasis HLB 200	6 mL MeOH	47–56%	40 (eff)	[64]
BTZ, 1MBZ, 4MBZ, 5MBZ	tap, effluent	Oasis HLB 200	10 mL EA	80–93%	ND	[44]
BTZ, 4MBZ, 5MBZ	airport runoff	Strata C-18 E 500	40 mL DCM	68–102%	0.3–10	[49]
BTZ, 1MBZ, 4MBZ, 5MBZ, 1OHBZ, 4OHBZ	recycled water	Oasis HLB 200 + Strata-X-AW(100)/ Strata-X-CW(100)/ Isoelute ENV+(150)	8 mL EA/MeOH/NH${}_{3}$ + 4 mL EA/MeOH/ HCOOH	ND	1-50	[60]
BTZ, 4MBZ, 5MBZ, DMBZ, ClBZ	wastewater	Strata-X 200	10 mL MeOH/ACN (1:1)	36–85%	52–376	[65,66]
BTZ, 5MBZ	river, influent, effluent	PLRP-s	ACN/H${}_{2}$O/HCOOH (24.9:74.9:0.2)	85–100% (eff)	0.8–1.1	[42,67]
BTZ	effluent	Bond Elut PPL 200	ND	ND	50	[55]
BTZ	tap, river, effluent	Oasis MAX (100) + Oasis MCX (100)	separate elution: MAX 6 mL MeOH/EA/HCOOH (69:29:2), MCX 6 mL MeOH/EA/NH${}_{3}$ (67.5:27.5:5)	93 ± 4%	5.7	[23]
2MBZ, 24DMBZ	groundwater, river	Merck EN 200	10 mL DCM	ND	3.3–6.7	[50]
BTZ, 4MBZ, 5MBZ, DMBZ, ClBZ	river, influent, effluent	Oasis MCX 500, Oasis MAX 500	MCX: 5 mL 5% NH${}_{3}$ in MeOH, MAX: 5 mL 5% HCOOH in MeOH	MCX: 87–105%, MAX: 75–92%	MCX: 5–17, MAX: 5–21	[38]
BTZ, 5MBZ	groundwater	PLRP-s	ACN/H${}_{2}$O/HCOOH (24.9:74.9:0.2)			[68]
BTZ, 4MBZ, 5MBZ, 1OHBZ, ClBZ	tap, lake, effluent	Poly-Sery HLB 60	2 mL MeOH	89–103%	0.4–4.8	[10]
BTZ	tap, recycled water	Oasis HLB 500	5 mL MeOH + 5 mL MTBE/MeOH (9:1)			[11]
BTZ, MBZ, DMBZ, ClBZ	influent, effluent	Oasis MCX 60	6 mL MeOH + 4 mL ACN	72–102%	500–2000	[58]
BTZ, MBZ	river	Oasis HLB 200	10 mL EA + 10 mL 0.1% NH${}_{3}$ in MeOH	31–72%	0.53–0.66	[41]
BTZ, DMBZ, ClBZ, NBZ	tap, swimming pool	Poly-Sery HLB 200	6 mL MeOH	95–105%	1.5–15	[69]
BTZ, MBZ	influent, effluent	Oasis HLB 200	5 mL 5% HCOOH in MeOH + 5 mL EA	108–154%	10–20	[70]
BTZ, 5MBZ	river, influent, effluent, aquaculture ponds	Oasis HLB	3 mL MeOH + 3 mL diethyl ether/MeOH (1:1)	91–104%	10	[71,72]
BTZ	river	Supel-Select HLB 200	6 mL MeOH/ACO/EA (2:2:1)	94%	ND	[73]
BTZ, MBZ	effluent	Oasis HLB 9 + Strata-X-AW(2.6)/ Strata-X-CW(2.6)/ Isoelute ENV+(3.8)	0.1% HCOOH in MeOH	97–102%	2–5	[61]
BTZ, 5MBZ, ClBZ, DMBZ	river	Poly-Sery HLB 200	6 mL MeOH	80–110%	0.4–1.5	[74]
BTZ, 4MBZ, 5MBZ, ClBZ, DMBZ	river	Oasis HLB 200 + Strata-X-AW(100)/ Strata-X-CW(100)/ Isoelute ENV+(150)	5 mL MeOH/ACN (2:8) + 6 mL 0.5% NH${}_{3}$ in MeOH/ACN (2:8) + 4 mL 1.7% HCOOH in MeOH/ACN (2:8)	22–112%	0.1-58	[54]
BTZ	wastewater	Oasis HLB 200 + Strata-X-AW(100)/ Strata-X-CW(100)/ Isoelute ENV+(150) + ENVI-Carb (200)	6 mL EA/ MeOH/NH${}_{3}$ + 3 mL EA/MeOH/ HCOOH	4–24%	ND	[56]
BTZ, 5MBZ, ClBZ, DMBZ	river, groundwater	Oasis HLB 500	3×2 mL MeOH/DCM (1:1)	71–95%	0.8–1.3	[57]

Oasis series is produced by Waters, Strata series by Phenomenex, Bond Elut by Agilent, Poly-sery by Anpel, PLRP-s by Spark, Isoelute ENV+ by Biotage, Supel-Select and ENVI-Carb by Supelco.

**Table 3:** SPE methods reported for benzotriazoles in solid samples (ND–no data).

Compounds	Sample Matrices	Cartidge Type, mg	Elution Solvent	Recovery	LOQ (ng/g)	Reference
BTZ, 5MBZ	detergent	Oasis HLB 500	5 mL 3% MeOH in DCM	ND	ND	[6]
BTZ, 5MBZ	river sediments, sludge	LSE (MeOH) + Oasis HLB 500	6 mL 15% MeOH in EA	70–226%	0.22	[43]
BTZ, 5MBZ, DMBZ, 1OHBZ	sludge, suspended particles	LSE (MeOH/H${}_{2}$O) + Strata-X 200	2×5 mL MeOH/ACN (1:1)			[39]
BTZ, 1MBZ, 4MBZ, 5MBZ	detergent, sludge	Oasis HLB 200	10 mL EA	80–93%	ND	[44]
BTZ, 4MBZ, 5MBZ, DMBZ, 1OHBZ, ClBZ	house dust	LSE (MeOH/H${}_{2}$O) + Oasis MAX 60	5 mL MeOH	71–108%	0.5	[15]
BTZ, 4MBZ, 5MBZ, DMBZ, ClBZ	sludge	PHWE + Oasis HLB 150 + Florisil 500	2×3 mL MeOH	39–89%	0.5	[75]
BTZ, 5MBZ	detergent	Oasis HLB 500	2×5 mL H${}_{2}$O (pH 2.9)/MeOH (95:5)	ND	ND	[22]
BTZ, 5MBZ	estuary sediments	ASE + Oasis HLB	6 mL MeOH	ND	1.5–1.8	[14]
BTZ, 4MBZ, 5MBZ, DMBZ, ClBZ	sludge	LSE (MeOH/H${}_{2}$O) + Strata-X 200	10 mL MeOH/ACN (1:1)	51–77%	118–1666	[65]
BTZ	plants	LSE (MeOH/H${}_{2}$O) + Oasis HLB 500	5 mL MeOH + 5 mL MTBE/MeOH (9:1)	100.5%	ND	[11]
5MBZ	soil	LSE (ACO/hexane) + Strata-X 100	10 mL EA	92–110%	0.002–0.019	[45]
BTZ, MBZ, DMBZ, ClBZ	sludge	LSE (MeOH/H${}_{2}$O) + Oasis MCX 60	6 mL MeOH + 4 mL ACN	ND	ND	[58]
BTZ, MBZ	sludge	LSE (MeOH) + Oasis HLB 200	5 mL 5% HCOOH in MeOH + 5 mL EA	97–9%	20	[70]
BTZ	sludge	MAE + Oasis HLB 250	5 mL MeOH + 5 mL MeOH/MTBE (1:9)	ND	ND	[76]

Oasis series is produced by Waters, Strata series by Phenomenex.

**Table 4:** SPE methods reported for benzotriazoles in human samples and industrial liquids (ND–no data).

Compounds	Sample Matrices	Cartidge Type, mg	Elution Solvent	Recovery	LOQ (ng/L)	Reference
BTZ, 4MBZ, 5MBZ	detergents; anti-icing, de-icing fluid	Bond Elut PPL 200	2 mL ACN/MeOH (1:1)	36–41% (anti-icing)	6–15	[52]
BTZ, 5MBZ, DMBZ, 1OHBZ	human urine	Oasis HLB 200	10 mL MeOH/ACN (1:1)	93–117%	0.2–5	[17]
BTZ	mineral oil	Sep-pak Plus 500	5 mL H${}_{2}$O/ACN (4:6)	77%	ND	[36]
BTZ, MBZ, 1OHBZ, ClBZ	human urine, amniotic fluid	Oasis HLB 200	10 mL MeOH/ACN (1:1)	urine: 67–106%, amn.fl. 71–93%	5–510	[18]

Oasis series and Sep-pak Plus are produced by Waters, Bond Elut by Agilent.

## Abbreviations

The following abbreviations are used in this manuscript:

ACN	acetonitrile
ACO	acetone
DCM	dichloromethane
EA	ethyl acetate
GC	gas chromatography
LC	liquid chromatography
LLE	liquid–liquid extraction
LSE	liquid–solid extraction
LOD	limit of detection
LOQ	limit of quantification
MeOH	methanol
MS	mass spectrometry
MTBE	methyl *tert*-butyl ether
